# Coverage of private sector community midwife services in rural Punjab, Pakistan: development and demand

**DOI:** 10.1186/s12961-015-0038-3

**Published:** 2015-11-25

**Authors:** Zubia Mumtaz, Adrienne V. Levay, Gian S. Jhangri, Afshan Bhatti

**Affiliations:** School of Public Health, University of Alberta, 3-309 Edmonton Clinic Health Academy, 1140 – 87 Ave, Edmonton, AB T6G 1C9 Canada; Real Medicine Foundation Pakistan, Islamabad, Pakistan

**Keywords:** Cost of care, Coverage, Private sector, Skilled birth attendant, Social determinants

## Abstract

**Background:**

In 2007, the Government of Pakistan introduced a new cadre of community midwives (CMWs) to address low skilled birth attendance rates in rural areas; this workforce is located in the private-sector. There are concerns about the effectiveness of the programme for increasing skilled birth attendance as previous experience from private-sector programmes has been sub-optimal. Indonesia first promoted private sector midwifery care, but the initiative failed to provide universal coverage and reduce maternal mortality rates.

**Methods:**

A clustered, stratified survey was conducted in the districts of Jhelum and Layyah, Punjab. A total of 1,457 women who gave birth in the 2 years prior to the survey were interviewed. χ^2^ analyses were performed to assess variation in coverage of maternal health services between the two districts. Logistic regression models were developed to explore whether differentials in coverage between the two districts could be explained by differential levels of development and demand for skilled birth attendance. Mean cost of childbirth care by type of provider was also calculated.

**Results:**

Overall, 7.9% of women surveyed reported a CMW-attended birth. Women in Jhelum were six times more likely to report a CMW-attended birth than women in Layyah. The mean cost of a CMW-attended birth compared favourably with a *dai*-attended birth. The CMWs were, however, having difficulty garnering community trust. The majority of women, when asked why they had not sought care from their neighbourhood CMW, cited a lack of trust in CMWs’ competency and that they wanted a different provider.

**Conclusions:**

The CMWs have yet to emerge as a significant maternity care provider in rural Punjab. Levels of overall community development determined uptake and hence coverage of CMW care. The CMWs were able to insert themselves into the maternal health marketplace in Jhelum because of an existing demand. A lower demand in Layyah meant there was less ‘space’ for the CMWs to enter the market. To ensure universal coverage, there is a need to revisit the strategy of introducing a new midwifery workforce in the private sector in contexts of low demand and marketing the benefits of skilled birth attendance.

## Background

Pakistan, with a maternal mortality rate (MMR) of 260 deaths per 100,000 live births [1], is one of six countries contributing to half of all maternal deaths worldwide [2]. A large body of historic and contemporary evidence from around the world has shown the introduction of an effective midwifery workforce is one of the most important elements of any maternal mortality reduction strategy.

Drawing on this well-acknowledged evidence, the Government of Pakistan introduced a new cadre of skilled birth attendants, the Community Midwives (CMWs) [3]. With the objective of providing skilled birth attendance to women living in rural areas, a large majority of whom prefer to give birth at home, the programme aimed to deploy 12,000 CMWs over a 5 year period between 2007 and 2012 [3]. Young, rural women who met recruitment criteria were given 18 months of midwifery training and were then deployed back to their home villages and expected to establish a private midwifery practice that provided care to a population of approximately 10,000. The CMWs were to establish home-based clinics and provide domiciliary antenatal care (ANC), childbirth, and postnatal care to rural women, as well as divert those currently using traditional birth attendants, or *dais*, to their skilled care. They were supported with a small stipend for the first 2 years after their training while establishing their practices. To date, over 8,000 CMWs have been deployed [3].

Concerns about the effectiveness of the programme for increasing coverage of skilled birth attendance in rural areas remain, particularly as the CMWs are located in the private sector. Historically successful midwifery programmes, such as ones from Sweden, England, Sri Lanka, and Thailand, have largely situated midwives in the public sector. The concept of locating a midwifery workforce in the private sector was first introduced by Indonesia in the 1980s; between 1989 and 1996, Indonesia deployed 54,000 midwives in as many villages in the country [4]. The trained midwives were provided with a government contract for 3 years and encouraged to develop a private practice with the expectation that they would gradually shift to a full private practice after 3–6 years [4]. Nearly 30 years later, the Indonesian MMR has stagnated at a high of 220/100,000 live births after an initial optimistic decline [5]. This stagnation is worrisome because MMR is one of the few health indicators that can be rapidly and consistently decreased. Malaysia and Sri Lanka halved their MMRs every 6–12 years between the 1950s and 1990s [6].

A large body of literature suggests that the Indonesian programme faltered because the midwives could not sustain their private practices after the expiration of their government contract [7-9]. Those who did manage to establish financially sustainable practices did so by serving wealthier clients, effectively excluding those who could not afford to pay their fees. According to the 2007 Indonesian Demographic Health Survey [10], 59% of poor women were still being attended by traditional birth attendants, while 80% of wealthy women were attended by nurses/midwives. This suggests the private sector model of midwifery care, with its inherent dependence on fee-paying clients, fails to provide universal coverage that is essential for the eradication of avoidable maternal deaths.

The design of the Pakistani CMW programme is similar to the Indonesian midwifery initiative. Like the Indonesian programme, the Pakistani CMWs are located in the private sector [3]. Further, recent evidence suggests that the Pakistani CMWs are also facing the same challenge in establishing private practices [11-13], given that young, under-confident women are perceived to have questionable competencies [12-14], resulting in a lack of trust from community women and their families [12,13]. Furthermore, young women cannot travel unhindered, a consequence of gendered norms that promote women’s seclusion in this context [11,13]; this limits the young CMWs’ ability to advertise their services and provide domiciliary care. Geographic distances, difficult terrain, and lack of transport infrastructure further exacerbate the CMWs’ ability to access clients [11].

Given the similarity of the Pakistani and Indonesian programmes, the question arises whether, 4 years after launch, the Pakistani CMW programme is expanding coverage of skilled birth attendance. Are there variations in uptake by demand for skilled birth attendants, which are in turn determined by overall levels of population education and socioeconomic status? Are CMW fees a factor in uptake of their services?

The present paper will address these questions using data from the districts of Jhelum and Layyah in Punjab; Jhelum being a relatively well-developed district [15], and Layyah one of the least developed [16]. The findings from this research will provide programme policymakers and managers with empirical evidence of the potential role of private sector skilled birth attendant programmes in contexts characterized by low levels of development.

## Methods

A cross-sectional, clustered, and stratified survey was conducted in two districts, Jhelum and Layyah, in the province of Punjab between November 2011 and July 2012. These districts were purposely selected because they were some of the first districts in which the programme was launched, they were safe enough for the researchers to collect data from, and span the range of socioeconomic development in Punjab. Historically, rain-fed agricultural practices in Jhelum could not sustain livelihoods, and men migrated to urban areas to find employment. This has led to overall higher levels of education and income levels in Jhelum. Layyah’s agriculture is based on a stable aqueduct irrigation system. The majority of residents are refugees settled here after migration from India in 1947, as well as a result of other natural disasters. The refugees were given small pieces of land that, because of the aqueduct, were sufficient to support families. It was postulated that these two districts would provide insight into the potential range of CMW coverage in the province, while acknowledging that the rates may not be representative of Punjab.

The study population consisted of women aged 15–49, who had given birth in the 2 years prior to the survey, and lived in CMW-served catchment areas (study clusters). The 2 year limit was used because the first batch of CMWs graduated in 2008. A total of 1,457 women were surveyed, 747 in Jhelum and 710 in Layyah. This sample size was calculated using the Pakistan Demographic Health Survey 2007 data [16], which showed that the probability of skilled birth attendance ranges from 0.25 in the  lower half of the socioeconomic index, to 0.5 in the  upper half of the index. This translates into an odds ratio (OR) of 3.0. To be conservative, we chose an OR of 1.8. In order to detect this difference with 80% power and 0.05 significance level, based on a logistic regression model of a binary outcome variable (skilled attendance at birth) on a binary independent variable (socioeconomic index), we estimated a minimum sample size of 880 women. This sample size was increased to 1,450 to take into account a multiple correlation between socioeconomic index and other predictor variables of 0.6 and a design factor of 1.3.

The sampling frame was drawn up using a three-stage stratified sample design. In the first stage, three out of four tehsils (sub-district units) in district Jhelum were randomly selected. All three tehsils in Layyah were selected. In the second stage, five union councils were randomly selected from each tehsil for a total of 30 union councils. If there was no CMW in a selected union council as indicated by programme records, it was replaced. In the third stage, two villages were randomly selected from each union council. However, if there were not enough eligible women in the selected village, a neighbouring village was included. This led to a total of 48 villages surveyed in Layyah and 69 villages in Jhelum. Within each selected village, a maximum of 22 women who gave birth in the 2 years prior to the survey were interviewed [17]. Given that about a quarter of the South Asian population is classified as low-caste and tend to be invisible, this group was oversampled [18,19]. We ensured that at least 11 of the 22 women interviewed in each village were landless and belonged to lower status castes. Lady health workers (LHWs) maintain records of births, deaths, and health status of all the members of households in their catchment populations. The women were surveyed in person by female enumerators. Using a pre-tested questionnaire, sociodemographic data, whether they sought ANC and from whom, place of delivery, type of birth attendant, and the amount spent on childbirth were explored.

Data were analyzed using Stata 11.0 [20]. Since low-caste women were oversampled, data was weighted on the basis that 25% of the population in South Asia is categorised as low-caste. All data was analyzed using 'svy' Stata commands to take into account both the weighting and stratified nature of the data. χ^2^ analyses were done to assess the sociodemographic characteristics of the sample population, use of ANC, type of ANC provider, type of birth attendant, and place of delivery stratified by district. Means were calculated for continuous data such as age, number of children, and cost of childbirth care. Logistic regression models using the forward selection approach were developed to find out if differentials in coverage between the two districts could be explained by differential levels of development and demand for skilled birth attendance.

Ethics approval was obtained from the University of Alberta Health Research Ethics Board and the National Bioethics Committee, Pakistan.

## Results

The mean age of our sample of 1,457 rural Punjabi women in Jhelum and Layyah was 28 years; 38% had received no schooling, almost all were currently married, with an average of 2.9 children. Overall, women in district Jhelum had higher levels of education, with twice as many women having 10 years of schooling compared to Layyah (37% vs. 19%). Women in Jhelum were wealthier, with only 18% classified as poor, compared to 33% in Layyah. Table 1 provides the sociodemographic characteristics of these women by district.

**Table 1:** **Sociodemographic characteristics of women included, overall and by district**

**Sociodemographic indicator**	**Overall (** ***n*** ** = 1457)**	**District**		***P*** **value**
		**Jhelum (** ***n*** ** = 747)**	**Layyah (** ***n*** ** = 710)**	
Age, mean ± SD	28.3 ± 0.15	28.0 ± 0.21	28.5 ± 0.22	0.99
Currently married, %	99.3	99.2	99.3	0.20
Mean number of children, mean ± SD	2.9 ± 0.05	2.7 ± 0.07	3.1 ± 0.08	1.00
Caste, %				0.00
High caste	45.4	59.3	30.8	
Middling caste	29.6	15.7	44.2	
Low caste (Kammis)	25.0	25.0	25.0	
Education, %				0.00
No education	38.2	24.5	52.7	
1–5 years schooling	26.9	30.0	23.7	
6–10 years schooling	28.1	36.9	18.6	
More than 11 years schooling	6.8	8.6	5.1	
Husband’s education, %				0.00
No education	20.7	14.3	27.5	
1–5 years schooling	14.9	11.2	18.7	
6–10 years schooling	50.8	62.2	38.8	
More than 11 years schooling	13.6	12.4	15.0	
Employment, %				
Yes	11.5	3.1	20.4	0.00
If employed, type of work (n = 199), %				0.00
Professional	10.3	18.2	9.0	
Skilled workers	67.0	68.2	66.9	
Agricultural labourers on other’s land	13.4	0	15.2	
Unskilled workers^a^	9.3	13.6	9.0	
Husband’s occupation, %				0.00
Professional/landowner	16.4	9.6	23.6	
Skilled worker	46.7	56.1	36.7	
Agricultural labourer on other’s land	5.4	0.9	10.0	
Unskilled worker	29.3	29.5	29.3	
Not working/unemployed	2.2	3.9	0.4	
Material asset index, %				0.00
First quartile (poorest)	25.9	18.7	33.4	
Second quartile	25.3	24.2	26.4	
Third quartile	24.0	27.7	20.1	
Fourth quartile (wealthy)	24.9	29.4	20.2	

^a^Domestic labour in people’s houses, at weddings and funerals.

### Use of CMW services

Overall, 47% of respondents knew of the presence of a CMW in their area, 62% in Jhelum and 38% in Layyah (*P* <0.001). Of these 47%, the majority knew that the CMW provided childbirth services and nearly 80% had had contact with her in the context of an introductory home-visit or provision of antenatal, postnatal, and general medical care(Table 2).

**Table 2:** **Women’s use of CMW services by district (%)**

**Maternal health service use indicator**	**Overall (** ***n*** ** = 1457)**	**District**		***P*** ** value**
		**Jhelum (** ***n*** ** = 747)**	**Layyah (** ***n*** ** = 710)**	
Sought at least one antenatal care (ANC) visit	96.6	97.8	95.3	0.01
Type of ANC provider				
Community Midwife (CMW)	8.3	13.8	2.4	0.00
Doctor	57.5	57.2	57.8	0.83
Skilled birth attendant	27.2	24.3	30.4	0.02
*Dai*	4.6	2.6	6.7	0.00
Type of childbirth attendant				
CMW	7.9	12.4	3.2	0.00
Doctor	36.1	47.6	24	0.00
Non-physician skilled birth attendant	25.5	26.3	24.6	0.49
*Dai*/traditional birth attendant	30.5	13.7	48.2	0.00
Place of delivery				
Home (home and *dai* home)	41.2	29.3	54	0.00
CMW clinic/home	1.1	3.1	0.7	0.01
Government	21.6	27.1	15.9	0.00
Private	35.1	40.5	29.4	0.00
Satisfaction with CMW care				
For ANC (n = 245)				0.05
Very satisfied	39.5	44.2	12.9	
Satisfied	53.7	50.0	74.2	
Somewhat satisfied	2.9	1.8	9.6	
Somewhat dissatisfied/dissatisfied	4.0	4.0	3.3	
For delivery (competency) (n = 109)				0.00
Very satisfied	68.1	75.2	39.3	
Satisfied	28.5	21.1	58.5	
Somewhat satisfied	1.7	1.6	2.2	
Somewhat dissatisfied/dissatisfied	1.7	2.2	0.0	

Values are presented as %.

CMWs have yet to emerge as a significant provider of maternal health services. Only 8.3% of women reported having received ANC from a CMW. This proportion is small given that uptake of at least one ANC visit is almost universal. Similarly, CMWs attended only 7.9% of all births. There were, however, noticeable differences by district. Nearly 14% of women in Jhelum reported receiving ANC from a CMW and 12.4% reported CMW-attended births. In Layyah, only 2.4% of women reported receiving ANC by a CMW and 3.2% reported a CMW-attended birth (*P* <0.001) (Table 2).

CMW home-clinics are a negligible child-birth facility with just 1% of all births occurring here. While reflective of the overall low levels of CMW care, the low rates also imply that the CMWs are providing domiciliary care; 77% of CMW-attended births took place in women’s own homes. Of the women who sought ANC from a CMW, 93% reported being satisfied or very satisfied with CMW care (Table 2).

Multivariate logistic regression showed that women in district Jhelum were six times more likely to report a CMW-attended birth compared to women in Layyah. This relationship strengthened significantly after adjusting for women’s age, education, their husband’s education, their occupation, husband’s occupation, and Material Asset Index. As seen in Table 3, none of these individual level sociodemographic characteristics had an independent relationship with a CMW-attended birth, but were included in the final model to control for important sociodemographic variables. The *P* value for the goodness-of-fit of the model was 0.151, indicating a good fit.

**Table 3:** **Odds ratios (OR) and 95% confidence intervals (95% CI) of CMW-attended births for women in Jhelum and Layyah**

	**Univariate**	**Multivariable**
Variable	OR (95% CI)	OR (95% CI)
District		
Layyah	1.0	1.0
Jhelum	4.27 (2.51–7.25)^*^	6.00 (3.26–10.9)^*^
Age		
15–19	1.0	1.0
20–29	0.40 (0.15–1.10)	0.50 (0.19–1.31)
30–39	0.38 (0.14–1.03)	0.42 (0.15–1.16)
40–49	0.34 (0.08–1.40)	0.32 (0.07–1.42)
Education		
No Education	1.0	1.0
1–5 years schooling	1.23 (0.74–2.03)	0.95 (0.53–1.69)
6–10 years schooling	0.81 (0.46–1.41)	0.49 (0.23–1.06)
More than 11 years schooling	0.17 (0.03–1.26)	0.09 (0.01–1.32)
Husband’s education		
No Education	1.0	1.0
1–5 years schooling	1.25 (0.66–2.37)	1.26 (0.63–2.56)
6–10 years schooling	0.68 (0.39–1.17)	0.57 (0.29–1.09)
More than 11 years schooling	0.55 (0.23–1.29)	0.86 (0.31–2.40)
Occupation		
Housewife	1.0	1.0
Professional	0.77 (0.15–3.93)	2.37 (0.43–13.1)
Skilled workers	0.35 (0.11–1.12)	0.68 (0.20–2.36)
Unskilled workers^a^	0.57 (0.08–4.20)	0.68 (0.08–5.67)
Husband’s occupation		
Professional/landowner	1.0	1.0
Skilled worker	1.43 (0.79–2.57)	1.15 (0.63–2.10)
Unskilled labour^a^	1.11 (0.60–2.07)	0.83 (0.43–1.60)
Not working	1.75 (0.90–3.41)	1.77 (0.88–3.52)
Material asset index		
First/quartile (poorest)	1.0	1.0
Second quartile	0.86 (0.49–1.51)	0.79 (0.42–1.47)
Third quartile	0.56 (0.30–1.04)	0.54 (0.26–1.13)
Fourth/non-poor	0.84 (0.47–1.50)	1.0 (0.47–2.13)

*P <0.01

^a^Domestic labour in people’s houses, at weddings and funerals.

### Cost of CMW services

A CMW-attended birth cost a mean of PKR 2950 (US $28; Table 4). This average masked the vast range of reported fees, from PKR 500–25,000 (US $5–240). The mean and the range are comparable with the cost of a *dai*-attended birth, which also ranged from PKR 200–25,000 (US $1.9–240). In contrast, a physician-attended birth cost, on average, PKR 13,000 (US $125) and ranged from PKR 200–60,000 (US $1.9–577). There was, however, a vast variation in physician-attended births in the public and private sectors, costing PKR 7,800 (US $75) and PKR 15,670 (US $151), respectively. Overall, 64% of CMW clients had saved money for their childbirth.

**Table 4:** **Cost of use of community midwife services by district**

	**Mean (95% CI) of PKR** ^**a**^			
**Amount of money spent on last delivery (PKR)**		**District**		***P*** ** value**
	**Overall**	**Jhelum**	**Layyah**	
Type of birth attendant				
CMW-attended birth	2944 (2315–3572)	3225 (2475–3976)	1760 (1000–2520)	0.03
Dai-attended birth	2254 (2059–2448)	3028 (2445–3612)	2015 (18407–2191)	0.00
Physician-attended birth overall	13025 (11927–14122)	14382 (1219–15846)	10299 (8871–11727)	0.00
Non-physician skilled birth-attendant birth	3996 (3579–4412)	4113 (3481–4745)	3834 (3355–43132)	0.26

^a^US $1 = PKR 103.

### Reasons for not seeking CMW care

Women who knew of a CMW and her services in their neighbourhoods, but had not sought care from the CMW, were asked their reasons for choosing not to do so (Table 5). The most common reasons given were that they preferred another provider, believed the CMW was not competent, did not trust her, or, in the case of ANC, did not know she provided said services. Importantly, geographic and social access, cost, marital status of CMW, and lack of CMW clinic/resources did not emerge as important barriers to the respondents’ uptake of CMW services.

**Table 5:** **Percent distribution of respondents’ cited reasons for not selecting a Community Midwife to provide antenatal care or attend delivery, overall and by district**

**Reason cited for not seeking**	**Overall (** ***n*** ** = 538)**	**District**		***P*** ** value**
		**Jhelum (** ***n*** ** = 304)**	**Layyah (** ***n*** ** = 234)**	
ANC from CMW				
Wanted ANC by other provider	24.1	25.1	23.2	0.61
CMW is not competent and is not trusted	22.1	24.8	19.7	0.16
Did not know she provided ANC	23.4	13.9	35.9	0.00
Not accessible geographically and socially	10.0	4.9	17.6	0.00
Respondent did not have transport	0	0	0	
She has no facilities	2.8	1.3	5.2	0.10
She is too expensive	1.4	2.3	0.4	0.07
She is unmarried	0.9	0	2.1	0.01
Childbirth attendance from CMW				
Wanted birth attended by other provider and CMW is not competent or trusted	16.1	25.0	6.0	0.00
She is not accessible socially or geographically	1.8	3.0	0.4	0.03
CMW clinic/house is uncomfortable/does not have necessary facilities	0.05	1.0	0	0.26
She is too expensive	1.1	1.7	0.4	0.24
She is unmarried	0.4	0	0.9	0.19

Respondents’ belief that CMWs lack competency is not supported by survey evidence. When comparing respondents’ reports of care received, those who had sought CMW services were as likely to receive elements of ANC at rates similar to those who reported care from physicians. These women were as likely to have a blood and urine test, receive two or more tetanus toxoid injections, and take iron supplements as women who received physician care. In fact, CMW clients were significantly more likely to have been weighed (OR = 1.66, *P* <0.05) and have had discussions about emergency birth-preparedness (OR = 2.3, *P* <0.001). They were, however, less likely to have had an ultrasound compared to women who sought care from a physician (OR = 0.16, *P* < 0.001).

An analysis of labour and childbirth care provided by the CMWs, as reported by the women, showed that CMWs remained present throughout labour and monitored it with regular vaginal examinations and fetal heart rates (100% and 95%, respectively). Over 90% of them used a safe delivery kit. Use of gloves for vaginal examinations was universal.

## Discussion

The objective of this research was to assess whether the CMW programme, located in the private sector, is expanding coverage of skilled birth attendance, and if any variations in this coverage are associated with demand and cost of CMW care. CMW coverage rates were compared in two distinctly different contexts, the relatively well developed Jhelum and the less developed Layyah, to assess if there were differences in skilled birth attendance between the two districts, and if these variations are reflected in CMW uptake rates. We assumed use of a skilled birth attendant indicated demand for such a provider. The results show that, overall, CMWs have yet to emerge as a significant maternity care provider in rural Punjab. Only 7.9% of all women who gave birth in the 2 years after deployment of a CMW in their neighbourhood reported a  CMW-attended birth. There are, however, large differences in coverage rates between the two districts, with women in Jhelum six times more likely to report a CMW-attended birth. The results also showed that the mean cost of a CMW-attended birth compared favourably with a *dai*-attended birth. Regardless, the CMWs were continuing to struggle in garnering community trust. The majority of women, when asked why they had not sought care from their neighbourhood CMW, cited a lack of trust in their competency and that they wanted a different provider.

One possible reason for low CMW coverage could be that the programme is relatively new and the CMW cadre relatively unknown; our results support this, as only 47% of respondents reported they knew of the CMW in their area. At the same time, 47% is not necessarily a small number and is not reflected in the low use rate of 7.9%, suggesting there may be other reasons for women not using their services besides a lack of awareness of CMW presence.

One of these reasons could be a lack of trust in CMWs competency, as this emerged as the most commonly cited reason for not seeking CMW services. The literature suggests trust and trustworthiness are key factors that determine the choice of a provider [21-23]. Lacking trust in providers has been shown to lead to lower patient satisfaction and withdrawal from their care [24,25]. While the importance of good interpersonal communication on the part of the provider is acknowledged for building trust, provider competence has been identified as the most important dimension of trust [23]. When patients perceive a provider as incompetent, they lose trust in the provider [26,27]. The present research assessed patients’ perception of CMW competence. Although it can be argued that a patient’s perception may not be a true reflection of actual provider competence, the literature suggests patient perception of lack of provider competency is itself a powerful determinant of trust [27]. In this case, however, their perception may be reflective of reality  as an emerging body of grey literature has identified issues with CMW competencies in Pakistan [12,14].

A second possibility for low CMW coverage is a lack of demand for skilled birth attendance. Assuming use equated demand, a use rate of 86% suggests a high demand for skilled birth attendance in Jhelum. In contrast, only 52% of women in Layyah reported skilled birth attendance, with the remaining 48% using *dais*. Use of CMWs was much higher in Jhelum versus Layyah (12.4% vs. 3.4% of all births). Taking these two findings together suggests the possibility that a greater overall demand for skilled birth attendance in Jhelum provided CMWs greater ‘space’ to insert themselves into the maternal health marketplace. In contrast, the lower demand for skilled birth attendance in Layyah meant there was less ‘space’ for the CMWs to enter the market. Ultimately, as indicated by a companion qualitative study, the issue in Layyah was not which type of skilled birth attendant to use, but whether to use one at all, as *dais* remained trusted and preferred providers [12].

Another closely related finding supporting the above possibility is that there is no variation in the sociodemographic characteristics of women who reported CMW use. Women, irrespective of levels of education or asset quartiles, are equally likely to report CMW care. Yet Jhelum still had higher proportions of women using their services than did Layyah. One interpretation is that it was the overall high level of development in Jhelum that promoted CMW use. In this context, women’s low levels of schooling was less of a determinant of CMW use than the context in which she lived. The results suggest that contextual factors may be as or even more important than individual factors – that typically  dominate the maternal health care literature – on skilled birth attendance and inequities. These findings are new in the skilled birth attendant literature, but have been reported previously in family planning literature [28].

It can be postulated that another reason for low use of CMW services is their cost. As discussed above, the CMWs are located in the private sector. Previous qualitative  work suggests that CMWs’ fees are a deterrent for potential clients [13]. Our survey data, albeit from different districts, shows that the average cost of a CMW-attended birth is relatively low, just slightly higher than the traditional birth attendants; this is a new finding from Pakistan. Research from Indonesia found midwives in Banten Province earned, on average in 2007, US $364 from their private practices [8]. While this amount does not reveal the specific amounts of fees charged for attending a birth, the authors describe it as a reasonably high income [8]. The low fees the Pakistani CMWs charge may, at one level, just be reflective of the fact that CMWs are nascent practitioners, attempting to establish their practices. Charging competitive rates might be a business strategy aimed at attracting clients away from *dais*. However, a companion qualitative study found that most CMWs were providing care to their relatives, who were charged nominal fees; they charged reasonably high fees, around PKR 6000 (US $58), for non-relatives [12]. The average was essentially skewed by their relatives’ use of their care.

## Conclusion

To summarize, the Pakistani CMWs have yet to emerge as a significant addition to the skilled birth attendant workforce in Pakistan. However, the variation in CMW coverage by development context of a district raises concerns that the CMWs' private sector location might translate into services only for those who can afford to pay while continuing to leave those women most vulnerable to maternal death unserved. This occurred in Indonesia and suggests a need to revisit the concept of private sector midwifery services.

## Abbreviations

ANC: Antenatal care
CMW: Community Midwife
MMR: Maternal mortality ratio
